# Joint Action Toxicity of Arsenic (As) and Lead (Pb) Mixtures in Developing Zebrafish

**DOI:** 10.3390/biom12121833

**Published:** 2022-12-08

**Authors:** Keturah Kiper, Jennifer L. Freeman

**Affiliations:** School of Health Sciences, Purdue University, West Lafayette, IN 47907, USA

**Keywords:** arsenic, developmental toxicity, lead, metal, mixtures, zebrafish

## Abstract

Arsenic (As) and lead (Pb) are environmental pollutants found in common sites and linked to similar adverse health effects. Multiple studies have investigated the toxicity of each metal individually or in complex mixtures. Studies defining the joint interaction of a binary exposure to As and Pb, especially during the earliest stages of development, are limited and lack confirmation of the predicted mixture interaction. We hypothesized that a mixture of As (iAsIII) and Pb will have a concentration addition (CA) interaction informed by common pathways of toxicity of the two metals. To test this hypothesis, developing zebrafish (1–120 h post fertilization; hpf) were first exposed to a wide range of concentrations of As or Pb separately to determine 120 hpf lethal concentrations. These data were then used in the CA and independent action (IA) models to predict the type of mixture interaction from a co-exposure to As and Pb. Three titration mixture experiments were completed to test prediction of observed As and Pb mixture interaction by keeping the Pb concentration constant and varying As concentrations in each experiment. The prediction accuracy of the two models was then calculated using the prediction deviation ratio (PDR) and Chi-square test and regression modeling applied to determine type of interaction. Individual metal exposures determined As and Pb concentrations at which 25% (39.0 ppm Pb, 40.2 ppm As), 50% (73.8 ppm Pb, 55.4 ppm As), 75% (99.9 ppm Pb, 66.6 ppm As), and 100% (121.7 ppm Pb, 77.3 ppm As) lethality was observed at 120 hpf. These data were used to graph the predicted mixture interaction using the CA and IA models. The titration experiments provided experimental observational data to assess the prediction. PDR values showed the CA model approached 1, whereas all PDR values for the IA model had large deviations from predicted data. In addition, the Chi-square test showed most observed results were significantly different from the predictions, except in the first experiment (Pb LC_25_ held constant) with the CA model. Regression modeling for the IA model showed primarily a synergistic response among all exposure scenarios, whereas the CA model indicated additive response at lower exposure concentrations and synergism at higher exposure concentrations. The CA model was a better predictor of the Pb and As binary mixture interaction compared to the IA model and was able to delineate types of mixture interactions among different binary exposure scenarios.

## 1. Introduction

Arsenic (As) and lead (Pb) are found ubiquitously in the environment as both naturally occurring metals in the earth’s surface and from industrial sources. Worldwide, it is estimated that 140 million people are exposed to drinking water contaminated with elevated As concentrations. In addition, in 2020 UNICEF reported that a third of the world’s children experience Pb poisoning. Furthermore, co-exposure to high levels of As and Pb has occurred at multiple global locations such as the East Chicago, IN USS Lead Superfund Site. Given As and Pb are both listed among the top ten chemicals of major public health concern by the World Health Organization, it is now more important than ever to build upon the individual chemical toxicological work to address the binary mixture exposures. Although, the specific toxicity mechanisms of a binary As and Pb mixture exposure is not yet known, there are common pathways that both metals target. As such, further work is needed to understand the type of mixture toxicity interaction that occurs.

Inorganic arsenite (iAsIII) toxicity occurs in multiple organ systems including the renal, vascular, and nervous systems and can accumulate in tissue [1]. As toxicity is primarily attributed to the imbalance of pro-oxidant to antioxidant ratio including increased protein oxidation and to the high affinity for thiol groups in functional proteins [2,3,4,5,6]. In most animals the bioactivation of iAsIII produces a plethora of intermediates and byproducts including reactive oxygen species (ROS) that can accumulate and lead to toxic effects, which is especially of concern considering As can cross the placental barrier and target the developing brain [7]. Furthermore, iAsIII undergoes detoxification through a metabolic pathway that produces other toxic As intermediate compounds including dimethylarsinic acid and methylarsonic acid before being excreted [8,9]. Laboratory animal studies with chronic sodium arsenite exposure (e.g., those with the zebrafish) report a significant induction of oxidative stress biomarkers and responses to antioxidants, specifically in brain tissues [10], supporting an increase in oxidative stress, which leads to apoptotic events [11]. While As is a known carcinogen, As exposure during development targets multiple biological systems including the cardiovascular and nervous systems [1,12,13]. Moreover, epidemiological studies show an association between urinary, hair, and toenail As concentrations with declined intelligence [14], impaired motor development [15], neuropsychological decline [16], and autism spectrum disorder [17]. In all of these studies, Pb was considered as a confounding variable since As and Pb are often found together in the environment. 

The heavy metal Pb is another major toxic metal with environmental sources stemming from naturally occurring processes and from improper waste management. Organic Pb does undergo metabolic processes in the liver unlike inorganic lead, to be excreted; however, its chemical properties are often confused for other essential molecules in the body enabling absorption of nearly all ingested organic Pb and unnecessary storage in cortical and trabecular bone, red blood cells, and soft tissues. In adults, Pb exposure via ingestion or inhalation can lead to a number of adverse health conditions [18], some of which can become permanent or lethal if left untreated. Developmental Pb exposure is associated with changes in uptake of essential metals and molecules in the brain [19], enhanced tauopathy and Alzheimer’s disease-like pathology [20], a reduction in tight junction molecules [21], and altered neuronal differentiation [22,23].

With widespread contamination and co-occurrence of As and Pb at global environmental sites, studies are beginning to address the mixture toxicity of these two metals but are almost always in a more complex combination with other metals or contaminants. For example, As and Pb in mixture with cadmium (Cd) and mercury (Hg) produced similar decreases in cell viability, increased apoptosis, ROS, and alterations in intracellular calcium [24]. In addition, developmental tertiary metal mixture studies with As, Pb, and Cd reported a significant down-regulation of GFAP(alpha) gene transcription, changes in cortical astrocyte cell death, and retarded skeletal growth [25,26]. While the exact mechanisms of toxicity initiated by exposure to a binary As and Pb mixture is not yet known, there are common pathways that both metals target including N-methyl-D-aspartate (NMDA) receptors [27], astrocytes [28], liver cells [29], and oligodendrocyte components [30]. As such, it is important to understand what type of mixture toxicity interaction occurs from the binary mixture exposure and to address this mixture interaction at multiple exposure scenarios.

Working to understand the toxicity of a singular toxicant is inefficient, but many of these studies have provided the field with important data needed to create in silico toxicity models. These in silico models predict the toxicity of mixtures providing an efficient and economical alternative to laboratory testing of all of the different toxicant mixtures. In general, mathematical models are used to predict concentrations at which no effects are observed or when a specific percentage of an effect will occur within a biological group including lethality or other binary outcomes [31]. Concentration addition (CA) and independent action (IA) models are used to determine if the components of a mixture experience joint action in an additive or independent manner, respectively. Each model has strengths and limitations and is important to investigate, which better represents the specific circumstances. Largely, an additive action indicates the effect of the chemical mixture is the sum of effects produced by similarly acting components, whereas the CA predicted effect can be interpreted in one of three ways: additive, synergistic, or antagonistic. IA can be plotted on the same graph and may set a limit for antagonism or indicate similar behavior between antagonism and independent action of the mixture components. IA is the product of a quantal response analysis, whereby the prediction of joint action assumes statistically independent distribution regarding recorded responses after toxicant exposure.

There is currently no published work evaluating developmental exposure during embryogenesis to early larval stages to a binary mixture of As and Pb. As mentioned above, As and Pb target similar systems in the body and cause toxicity through some similar mechanisms associated with ROS; therefore, we hypothesized that in mixture these two metals interact and can be modeled by CA. To address this research question, the zebrafish was applied as a robust vertebrate model for the investigation of developmental metal mixture toxicity [32,33,34,35,36,37,38,39]. The zebrafish is currently used for studying mixture toxicity [31,40], highlighting advantages in the need for only small quantities of chemical mixture volumes in well or Petri plate exposure vessels and availability of large numbers of embryos for use in studies determining joint interaction mixture models. In addition, single metal toxicity studies with As and Pb support similar toxicity outcomes [6,11,13,37,38,39,41,42,43,44,45,46,47,48,49,50,51]. Consequently, this study is centered around the collection of individual toxicity data of each metal, the determination of an additive mixture effect, and the comparison of the predicted outcome of the CA and IA models with the observed outcomes.

## 2. Materials and Methods

### 2.1. Animal Husbandry

**Figure 1:**
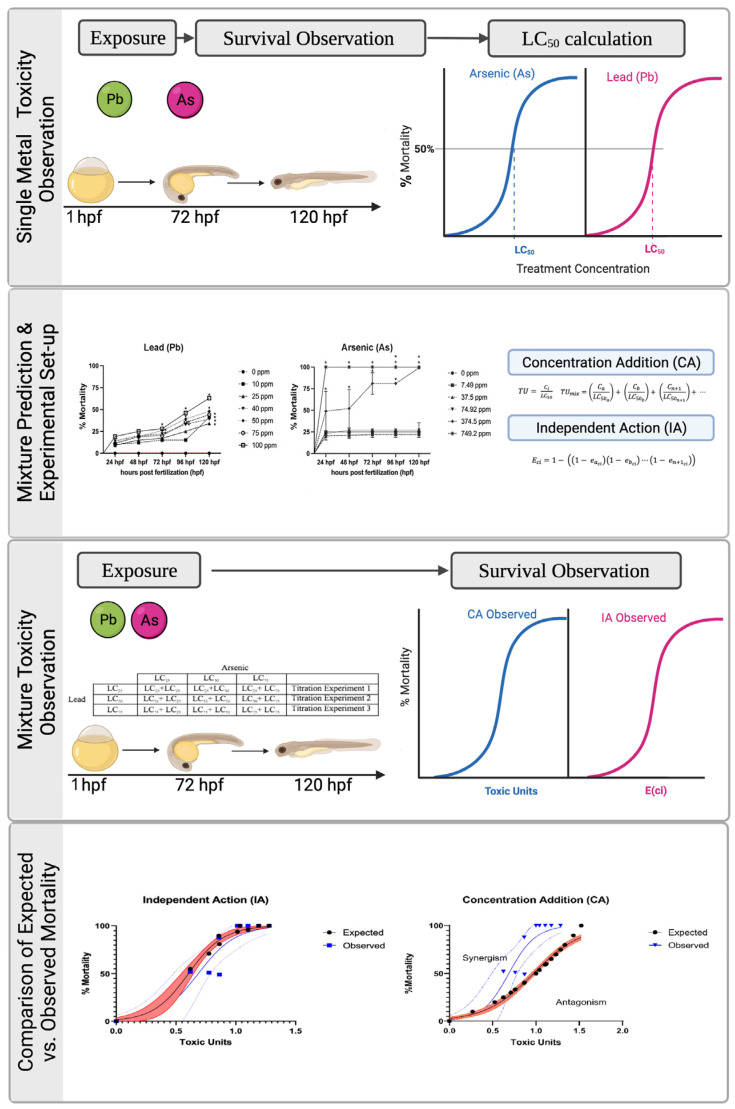
**Experimental overview of study.** Zebrafish embryos were dosed with a concentration range of each metal to determine lethal concentrations (LC) for benchmark treatment groups in the mixture titration experiments and for predictive mathematical modeling. The expected and observed results were compared among the mathematical models using the prediction deviation ratio and Chi-square test. Type of mixture interaction was determined using regression modeling.

Wild type adult zebrafish of the 5D strain were maintained at 28 °C under a light: dark cycle ratio of 14:10 h Adult zebrafish were kept in an open flow system with pH between 7.0–7.3 and salinity at 550–600 µS/cm conductivity. The adult zebrafish were maintained, fed, and bred according to established protocols [52]. Embryos were obtained by placing adults in spawning tanks with embryo collection occurring immediately after fertilization; this is completed with ease as zebrafish have photoperiodic nature with mating triggered by sunrise or natural lighting in a laboratory setting. Embryos used in this study were collected between the 2–16 cell stage, assessed for lack of coagulation of fertilized eggs, and exposed to metal treatments for further experimental procedures. Zebrafish embryos are within a chorion until hatching. Past studies demonstrate that most chemicals including metals can easily pass through the chorion to be absorbed in embryonic tissue [39]. As such, removal of the chorion is not necessary for studies assessing the toxicity of As or Pb prior to hatching and we followed similar exposure paradigms as our past Pb studies [33,36,37,38,39]. All protocols were approved by the Purdue University Institutional Animal Care and Use Committee (PACUC) with all fish treated humanely and aligned to alleviate suffering. The overall experimental design is summarized in Figure 1.

### 2.2. Arsenic and Lead Single Chemical Exposure

To determine the lethal concentrations (LC) at 25%, 50%, and 75% (i.e., LC_25_, LC_50_, LC_75_, respectively) for the larval population, acute toxicity tests were performed. Zebrafish embryos were treated with sodium meta arsenite (>90% purity, CAS 7784-46-5; Sigma Aldrich, St. Louis, MO, USA) or Pb acetate (99.99% purity, CAS 6080-56-4; Sigma-Aldrich). Zebrafish embryos were continuously exposed to 0–749.13 parts per million (ppm, mg/L) (0–10 mM) of As at a pH of 7.2 or 0–100 ppm (0–0.48 mM) of Pb at pH of 7.2 from 1 to 120 h post fertilization (hpf). Concentrations of dosing solutions were confirmed to be within expected concentrations using US Environmental Protection Agency approved water test kits (As: Industrial Test Systems; Pb: Osumex). In addition, the highest test concentration used in the Pb toxicity assessment aligned with our past study confirming solubility as Pb acetate at these concentrations (i.e., up to 100 ppm) [37]. Negative controls were treated with filtered fish water to match solutions in which metals were dissolved. Four biological replicates (N = 4) of each treatment group each comprised of either As or Pb treatments with 50 subsamples in a Petri dish were exposed and mortality was recorded every 24 h. A light microscope was used, starting at 24 hpf, to detect the presence of a heartbeat. The absence of a heartbeat was defined as dead. All dead larvae were removed to determine the mortality count at each 24 h period.

### 2.3. Single Arsenic and Lead LC Calculations

For each treatment group in each replicate, the mortality (M) of the zebrafish larvae in each treatment group (T_i_) was calculated as a ratio of the total number of larvae with a heartbeat (x_alive_) to the total number of zebrafish embryos treated at 0 hpf (x_total_)

M (T_i_) = [x_alive_/x_total_]_i_ (1)

The percent mortality of zebrafish larvae was then normalized to represent the relative mortality (RM) of a treatment group (T_i_) to the control; this is represented by the ratio of percent mortality of the zebrafish larvae in the treatment group M(T_i_) to the mortality of the negative control M(T_control_)

RM (T_i_) = [M(T_i_)/S(T_control_)] × 100 (2)

The mean mortality of the four replicates for each treatment group was calculated from the relative mortality of each replicate. The mean proportion of dead larvae at each time point was calculated for each concentration. The different lethal concentrations of the single components predicted to elicit a specific response were found using excel. The concentrations at which As or Pb were predicted to have 0%, 25%, 50%, 75%, and 100% lethality was calculated. In addition, an analysis of variance (ANOVA) with SAS software (version 9.2; SAS Institute Inc., Cary, NC, USA) by developmental time point was performed to determine significant differences among the treatment concentrations. Means were compared using the least significant difference test at α = 0.05 when a significant ANOVA was observed.

### 2.4. Binary Arsenic and Lead Mixture Exposure

The mixture concentrations selected for joint action modeling were determined from the single chemical LC_50_ results. Unlike the single chemical toxicity assessments, preliminary laboratory studies indicated precipitation of Pb in the mixture solution at a neutral pH at high concentrations as confirmed with water testing kits (i.e., As concentrations were as expected, but Pb was at a lower than expected concentration). To avoid precipitation, pH of the dosing solution was lowered to 6.5. Our past study has shown that there is no effect on mortality at a pH of 6.5 in developing zebrafish [53]. Specifically, an increased mortality is not observed until pH is less than 3.5, supporting dosing solutions at pH of 6.5 would not confound metal toxicity. Subsequently the pH of the LC_50_ mixture treatment solutions was decreased to 6.5 for these experiments. Water testing confirmed at this pH metal concentrations were within expected range (i.e., Pb was no longer precipitating) and no change in mortality was observed in the negative control treatments (0 ppm) at pH 6.5. 

The titration method was used to generate the mixture combinations. In the titration method the concentration of one metal (in this study Pb) was held constant, while the other metal concentration (in this study As) was increased in each additional treatment group. This experiment was repeated two more times, but with an increased concentration of Pb (i.e., at the LC_25_, LC_50_, and LC_75_) for each additional titration experiment with As (Table 1). The values collected from the individual chemical are represented in the results section in Table 2. Three biological replicates (N = 3) with 50 subsamples in a Petri dish were exposed to binary mixture treatments and mortality was recorded every 24 h. A light microscope was used, starting at 24 hpf, to detect the presence of a heartbeat. The absence of a heartbeat was defined as dead. All dead larvae were removed to determine the mortality count at each 24 h period. We performed an ANOVA with SAS software (version 9.2; SAS Institute Inc., Cary, NC) by developmental time point to determine differences among the treatment groups. Means were compared using the least significant difference test at α = 0.05 when a significant ANOVA was observed.

### 2.5. Independent Action (IA) and Concentration Addition (CA) Model Calculations

Different lethal concentrations of the individual metals were obtained using polynomial regressions for the calculated and bimodal observed mortality. An x-axis unit was calculated to scale the individual concentrations of As and Pb to predict the mortality outcome of a binary mixture treatment. Toxic units (TU) were calculated by dividing the concentration of the individual metal, Ci, to its corresponding LC_50_.

For the concentration addition model, the following equations were used:

TU = C_i_/LC_50_ (3)

C_i_ is the concentration of the individual metal *i* and the LC_50_ of that of metal *i*. 

In the CA method, the TU_mix_ (x-value) is graphically related with the LC_25_, LC_50_, and LC_75_ (y-axis) of As and Pb added together to create the x-values needed for the predicted mixture LC_50_ graph. For the CA model, the TU of a mixture was

TU_mix_ = (C_a_/LC_50a_) + (C_b_/LC_50b_) + (C_n+1_/LC_50n+1_)+ ⋯ (4)

The above equation was used to model the predicted mortality of each metal at the same predicted level of lethality. For example, the LC_10mix_ = LC_10As_ + LC_10Pb_. Once these values were determined they were graphed and line of fit was calculated. Next the TU for the mixtures were plugged into the function produced in the regression run on the CA predicted mortality graph. The y value returned for each TU_mix_ represents the % mortality expected. 

In the IA model

E_ci_ = 1 − ((1 − e_aci_) (1 − e_bci_)⋯(1 − e_n+1ci_)) (5)

where the expected response for a mixture, E (x1,2…n) equals to the product of each chemical’s probability of no effect (1–E) subtracted from 1. In the IA model, the concentration selected for chemicals in the mixture must have the same predicted individual mortality as this model proposes the combination of the chemicals not resulting in an additive toxicity effect.

### 2.6. Mixture Toxicity Analysis and Comparison of Measured with Predicted Toxicity

To determine the accuracy of these predictive models, the observed toxicity response was compared to the predicted response by calculating the prediction deviation ratio (PDR).

PDR = (y_i_/x_i_) = (EC_predicted_/EC_observed_) (6)

PDR represents the relative distance of an observed value to its predicted counterpart, where x_i_ is the observed value and y_i_ is the predicted value. Therefore, a correct estimation is represented by a PDR of 1. An underestimation of the toxicity is represented by a PDR < 1 and an overestimation represented by a PDR > 1. In addition, the log (PDR) values were calculated and plotted for the equivalent LC mixture to compare the predictive value of the CA and IA models (i.e., was the prediction underestimating or overestimating the response as indicated by log (PDR) < 0 or >0, respectively). A Chi-square test was also used to compare the observed mortality and the predicted mortality from the IA and CA model at alpha = 0.05 for significance.

### 2.7. Type of Mixture Interaction Analysis 

The observed mortality responses of the mixture treatments were plotted on the same graph with a 3rd order polynomial as the expected effect line to determine the type of mixture interaction for As and Pb. Reference boundaries for interactive effects was implemented using 95% confidence intervals (CI) for the predicted model curves to determine additive, synergistic, or antagonistic interaction. Residual plots were then used to determine if a polynomial regression was appropriate for the mortality data collected on individual chemicals. The LC_50_ and the confidence limits (95%) were determined using GraphPad Prism version 8.0.0.

## 3. Results

### 3.1. Effects of Single Chemical Exposure on Mortality

**Figure 2:**
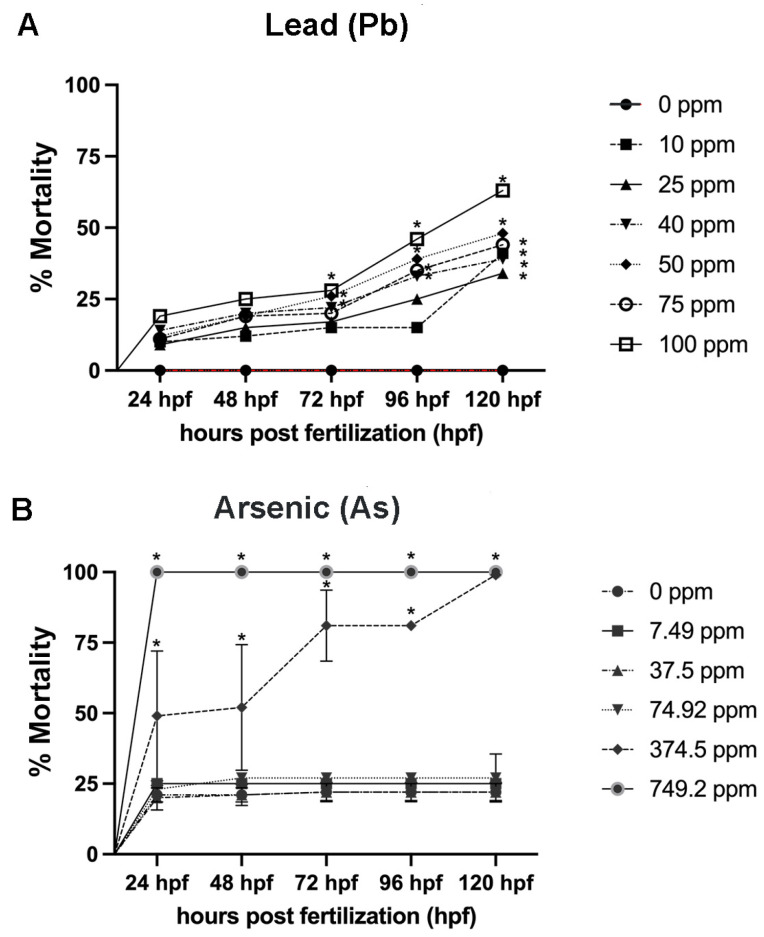
**Percent mortality assessed every 24 h from 1–120 h post fertilization (hpf) for Pb and As.** Single chemical exposures for Pb (**A**) and As (**B**). N = 4 biological replicates for each treatment group with 50 subsamples per each treatment group. Data is presented as mean ± standard deviation. * *p* < 0.05.

Developing zebrafish were exposed to a range of concentrations of Pb [0–100 ppm] or As [0–749.2 ppm]. For the Pb treatments, no significant impacts on mortality were observed at 24 or 48 hpf (Figure 2A) (*p* > 0.05). At 72 hpf, a significant increase in mortality was observed in the three highest treatment groups (50, 75, and 100 ppm), at 96 hpf the four highest treatment groups (40, 50, 75, and 100 ppm), and at 120 hpf in all treatment groups (Figure 2A) (*p* < 0.05). There was limited variability among the replicates (as indicated by limited error bars). For Pb, the 120 hpf-LC_25_ was 39.0 ppm, the 120 hpf-LC_50_ was 73.76 ppm, and the 120 hpf-LC_75_ was 121.74 ppm (Table 2).

**Table 2:** Lethal concentrations of single metal exposure to arsenic (As) or lead (Pb) at 120 h post fertilization (hpf) in parts per million (ppm, mg/L).

Chemical	Percent (%) Mortality				
	0%	25%	50%	75%	100%
**Lead (Pb)**	0 ppm	39.0 ppm	73.76 ppm	99.94 ppm	121.74 ppm
**Arsenic (As)**	0 ppm	40.18 ppm	55.42 ppm	66.64 ppm	77.32 pm

In the As treatment groups, there was more variability observed among the replicates, but the highest concentrations (374.5 and 749.2 ppm) showed a significant increase in mortality at all developmental time points (Figure 2B) (*p* < 0.05). The treatment group of 74.92 ppm As and all lesser treatment groups did not show significant differences in mortality rates compared to the negative control at any developmental time point (Figure 2B) (*p* > 0.05). As a result, the 120 hpf-LC_25_ was 40.18 ppm, 120 hpf-LC_50_ was 55.42 ppm, and 120 hpf-LC_75_ was 77.32 ppm for As (Table 2).

### 3.2. Effects of Metal Mixtures on Mortality

**Figure 3:**
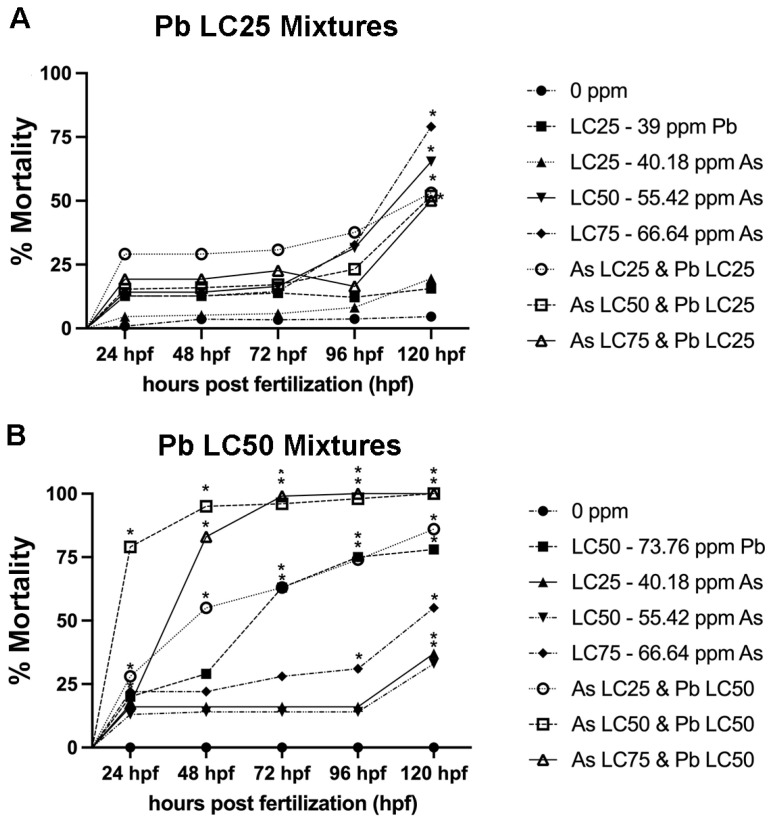
**Percent mortality for mixture experiments assessed every 24 h from 1–120 h post fertilization (hpf).** Mixture experiments in which Pb was held constant at the 120 hpf-LC_25_ (39.0 ppm) in experiment 1 (**A**) and in which Pb was held constant at the 120 hpf-LC_50_ (73.76 ppm) in experiment 2 (**B**), while As was varied to include the 120 hpf-LC_25_, 120 hpf-LC_50_, and 120 hpf-LC_75_. N = 3 biological replicates for each treatment group with 50 subsamples per each treatment group. Data is presented as mean ± standard deviation. * *p* < 0.05.

Experiment one of the titration series kept the Pb concentration treatment consistent at the 120 hpf-LC_25_ (39.0 ppm), while As concentration varied at the 120 hpf-LC_25_, 120 hpf-LC_50_, and 120 hpf-LC_75_ (Table 1). At 120 hpf, mortality in all mixture treatment groups and the As LC_50_ and As LC_75_ was significantly decreased compared to the control (*p* < 0.05) (Figure 3A). 

In the second experiment of the titration series again the Pb concentration treatment was held consistent, but at the 120 hpf-LC_50_ (73.76 ppm) and the same As concentrations included as in experiment 1 (Table 1). There was a significant increase in mortality in all mixtures at 24 and 48 hpf (*p* < 0.05) showing a dose response, while all other treatment groups showed no significant difference from the negative control (Figure 3B) (*p* > 0.05). At 72 hpf, all three mixtures and the Pb LC_50_ were statistically different from the control (*p* < 0.05) (Figure 3B). By 96 hpf, the As LC_75_ treatment group was also significantly different from the control and at 120 hpf all treatment groups had significantly increased mortality compared to the control (*p* < 0.05) (Figure 3B). 

In experiment 3, there was 100% lethality for all mixtures at the Pb 120 hpf-LC_75_ (99.94 ppm) by 24 hpf. As such, experiment 3 stopped early as the mortality was at 100% for mixtures once the titration of Pb LC value increased. This means that for both the CA and IA models there are data missing based on this observation.

### 3.3. Mixture Toxicity Comparison of Predicted to Observed Toxicity 

**Figure 4:**
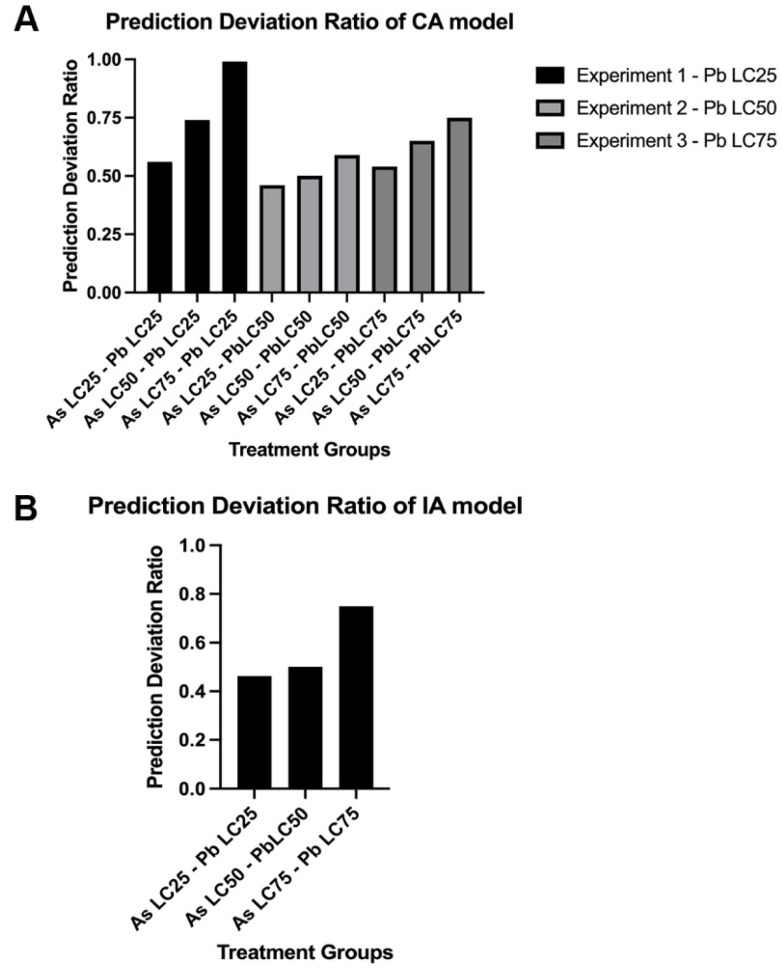
**Comparison of prediction deviation ratio (PDR) of mixtures for the CA and IA models.** For the CA model (**A**), each exposure scenario in each experiment was evaluated for PDR values that approached 1. The IA model (**B**) is limited to evaluating metal mixtures at the same LC percentage for approaching 1. N = 3 biological replicates for each treatment group with 50 subsamples per each treatment group.

PDR values were calculated for each exposure experiment for the CA model to assess prediction of the model (i.e., for approaching 1) (Figure 4A). In all three experiments, the Pb concentration with As LC_25_ was near 0.50, but as the As concentration increased (i.e., higher LC percentage) the PDR was closer to 1. Experiment 1 (Pb LC_25_ held constant) was the best prediction with values approaching 1, while experiment 2 (Pb LC_50_ held constant) was the furthest from 1. For this assessment the IA model data could only be analyzed at equal LC percentages (i.e., LC_25_ for As and Pb, LC_50_ for As and Pb, and LC_75_ for As and Pb) (Figure 4B). As metal concentrations increased the model better predicted toxicity, but the best PDR was only near 0.8 for the LC_75_ mixture.

**Figure 5:**
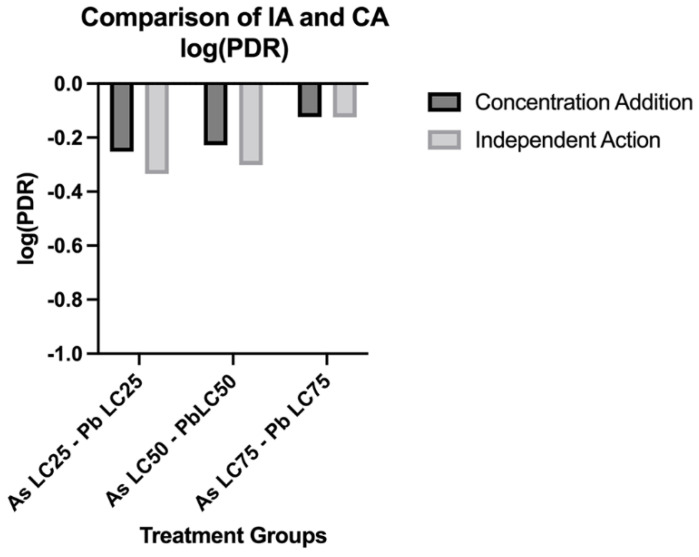
**Comparison of predicted lethality using the Independent Action (IA) and Concentration Addition (CA) models.** The log of the prediction deviation ratios (PDR) of mixtures for the CA and IA models. N = 3 biological replicates for each treatment group with 50 subsamples per each treatment group.

The log(PDR) was then calculated to determine whether the CA and IA models over- or underestimated mortality. In both models, mixture treatments at the same LC percentages have underestimated toxicity with all log(PDR) values < 0 compared to observed toxicity (Figure 5). 

The Chi-square test indicated the CA model was not significantly different from the observed data in experiment 1 (Pb LC_25_) for the As LC_50_ and LC_75_ mixtures (*p* > 0.05); however, the observed data was different for the As LC_25_ mixture (*p* = 0.0001). In addition, all mixture treatments in experiments 2 and 3 were significantly different between the predicted mortality and the observed (*p* < 0.0001) (Table 3). Application of the Chi-square test on the IA predicted compared to observed mortality also revealed a significant difference in the IA model predictions and the observed mortality (*p* = 0.0001).

### 3.4. Type of Mixture Interaction Analysis 

**Figure 6:**
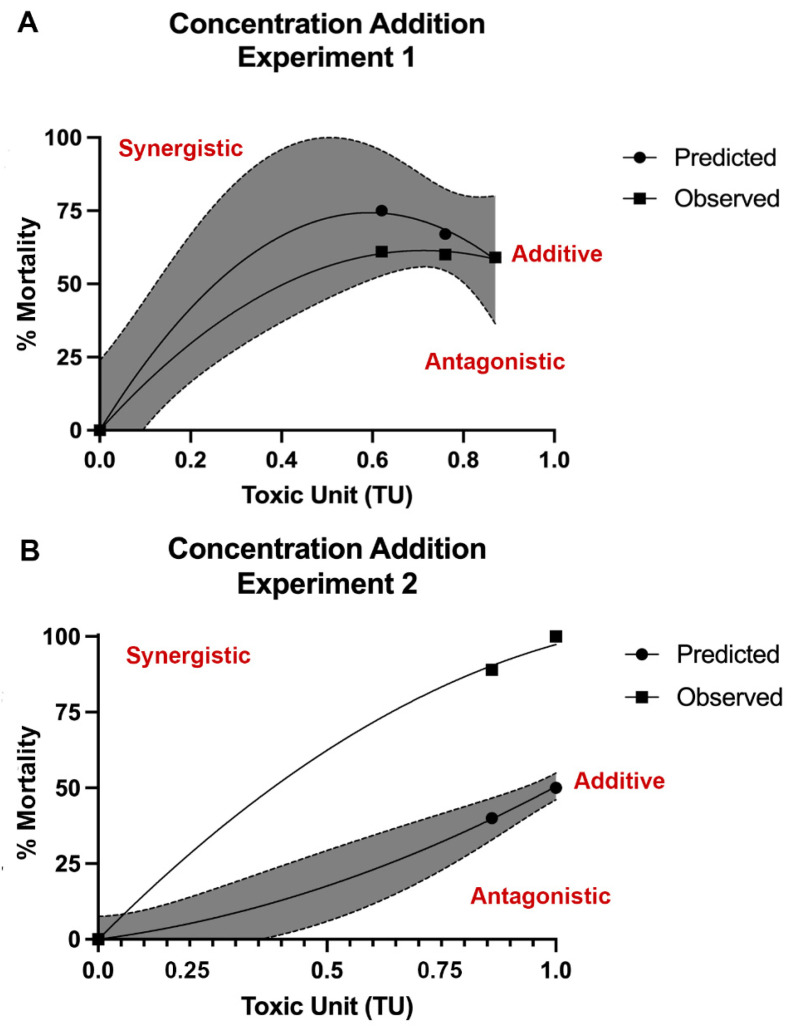
**Observed and predicted mortality using the concentration addition (CA) model.** Developing zebrafish were exposed to the metal mixtures from 1 to 120 h post fertilization (hpf) and mortality recorded. Results were compared to predicted mortality based on CA models when the Pb LC_25_ (39.0 ppm, mg/L) concentration was held constant (**A**) or when the Pb LC_50_ (73.76 ppm) concentration was held constant (**B**). Similarity of predicted and observed curves indicates additive interaction (within in shaded region) (**A**), where upper deviation from predicted curve suggests synergistic response (**B**). N = 3 biological replicates per treatment group with 50 subsamples per biological replicate.

Further analysis was completed to determine the type of mixture interaction. We hypothesized a potential synergistic reaction may be occurring, which would explain underestimation of mortality observed for mixture treatment exposures from PDR calculations. The mixture treatment experimental data was plotted on the same graph with the predicted curve attained from the mathematical modeling of the single metal experiments. For the CA model, experiments 1 and 2 were plotted separately. Experiment 3 could not be assessed given 100% mortality by 24 hpf in the mixture treatment groups. In experiment 1 with Pb LC_25_ constant, an additive mixture interaction was indicated based on similarity of curves (Figure 6A). Alternatively, in experiment 2 with the Pb LC_50_ held constant, a synergistic mixture interaction was suggested based on upper deviation of observed lethality (Figure 6B). It is hypothesized that a synergistic response also occurred in experiment 3 with Pb LC_75_ held constant given increased mortality in experimental observations (i.e., 100% mortality by 24 hpf in all mixture treatment groups) compared to what was predicted. 

**Figure 7:**
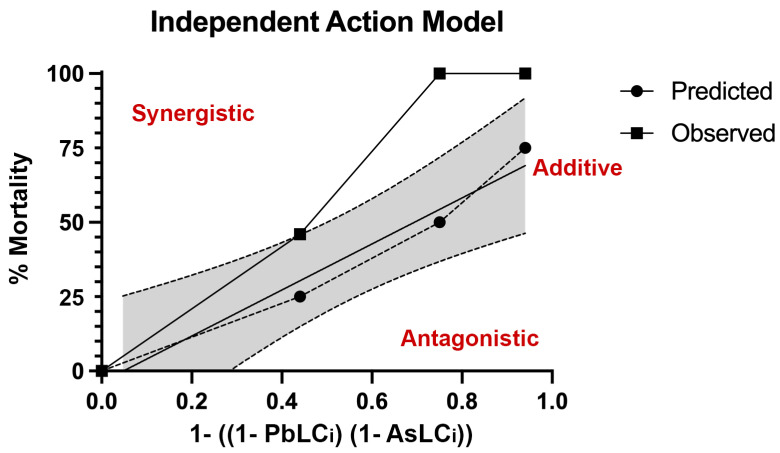
**Observed and predicted mortality using the independent action (IA) model.** Developing zebrafish were exposed to the metal mixtures from 1 to 120 h post fertilization (hpf) and mortality recorded. Results were compared to predicted mortality based on the IA model, where the predicted LC of each metal is equal. The upper deviation of the observed curve compared to the predicted curve suggests a synergistic response. N = 3 biological replicates per treatment group with 50 subsamples per biological replicate.

Given limitations of the IA model, assessments could only be completed when the LC was the same for each metal similar to the PDR and Chi-square analysis. Overall, a synergistic response is suggested given upper deviation from predicted curve (Figure 7).

## 4. Discussion

From a global perspective environmental contamination of heavy metals like As and Pb continues to grow and in tandem the general population’s exposure can increase. This study defined the impact of a developmental exposure to a binary As and Pb mixture using joint action models. In addition, the predicted models were tested for their accuracy based on additional mixture toxicity observational assessments. Overall, lethal concentrations observed in the single metal exposure studies agreed with previous findings under similar conditions with developing zebrafish. For instance, a study exposing zebrafish to As from 4–120 hpf reported that from 72–120 hpf there was also 100% mortality at concentrations above 112 ppm As, which agrees with the observed individual As mortality data collected in this study [54]. The LC_50_ of As determined in our study was 55.42 ppm, which is within the range found in other acute toxicity studies using the developing zebrafish [48]. In addition, after 120 h of developmental exposure, zebrafish larvae had ~12–16% mortality rate at ~38 ppm [49], supporting that 25% mortality can occur at 40 ppm As as found in our study. For Pb, a past laboratory study reported that over 50% mortality was observed at 120 hpf at 50 and 100 ppm exposure concentrations, which is also in agreement with the data reported here [36,37]. Overall, the single metal mortality data predominately agrees with other studies of developmental As or Pb exposures using the same biological model. However, there are occasions in the literature where a lower Pb concentration was recorded as being responsible for 50% lethality [55].

This study focused on determining if As and Pb mixtures may have a joint action, and if so, could this joint action be represented with the CA or IA models. Overall, both models underestimated mortality of these two metals given the concentrations used. The CA model was a better predictor for the mixture points included in both the CA and IA analysis given the PDR values more closely approaching 1 but overall, almost all observed mortality was statistically different than the expected mortality based on the Chi-square test. The only exception was two of the treatment groups in experiment 1 where the Pb LC_25_ was held constant. Moreover, the type of mixture interaction was also different in experiment 1 of the CA model (additive) compared to that of experiment 2 of the CA model and the IA model (synergistic). It is also hypothesized that a synergistic response occurred in experiment 3 where the Pb LC_75_ was held constant given that embryos were dead in all mixture treatment groups by 24 hpf, which did not follow the prediction. A synergistic response provides rationale as to why the models underestimated toxicity and why almost all observed toxicity for the mixture treatment groups was statistically different than what was predicted. 

In the literature there are several factors reported to influence the successful application of the joint action models. The type of toxicant (metal, pesticide, etc.), the model (vertebrate versus invertebrate), distribution of data points, and the number of mixtures data points that are input into these models. The CA model in the context of metal mixtures and pesticide mixtures has been found to have the best predictions of joint interaction within ecotoxicology [56]. Of the 202 pesticide mixtures evaluated, 90% of observed effects fell within a factor of 2 of the predicted values for the CA model [56,57]. However, the CA model does fall short in predicting the observed data in 75% of metal mixtures that had Zn, copper, and Cd [58]. Although chemical mixture studies are increasing, there are still questions regarding how to best incorporate mixture modeling into developmental toxicity research including studies using the zebrafish and other animal models. For example, an in silico study found that at low effect concentrations, the CA model overestimates metal mixture toxicity by a factor of 1.2 when considering a range of species responses. Using this same in silico platform, it was determined that the best use of these models in prediction is in a tiered assessment process, where the CA model is the first tier as it is more conservative in predictions compared to the IA model [59]. This is different than the response observed in the present study where the CA model underestimated toxicity. 

Calculating the PDR or using statistical models are helpful tools that highlight the statistical relevance of deviations from the predicted joint actions of metal mixtures, but studies report some irreproducible data [60]. A reason for the inconsistent deviations from the predicted additivity can be varying isobologram shaped response or as observed in this study, a synergistic response. Using these models and statistic-dependent approaches can be an origin for further studies. Overall, this study indicates that Pb and As produces a joint action response that was confirmed with observational studies. The CA model was a better predictor of the observed response and revealed an additive response in one exposure scenario and a synergistic response in another, whereas given limitations of the IA model only a synergistic response is able to be seen. The difference in the type of mixture interaction observed in the CA model suggests the mixture interaction is dependent on severity of effect and demonstrates need to apply multiple joint action models in mixture toxicity studies. 

Future studies are needed to further assess the Pb and As mixture interaction for additional toxicity outcomes to compare type of mixture interaction observed. In addition, the current study included higher Pb and As concentrations to induce the measured effect (i.e., LC_25_, LC_50_, and LC_75_) with Pb 120 hpf-LC_25_ of 39.0 ppm and As 120 hpf-LC_25_ of 40.18 ppm. While these concentrations are reported in soil and water in highly contaminated areas, additional studies addressing lower exposure concentrations more similar to what is observed amongst global populations today are needed. 

## Figures and Tables

**Table 1:** Lead (Pb) and arsenic (As) mixture titration experimental design.

Experiment	Mixture 1	Mixture 2	Mixture 3
**Experiment 1**	Pb LC_25_ + As LC_25_	Pb LC_25_ + As LC_50_	Pb LC_25_ + As LC_75_
**Experiment 2**	Pb LC_50_ + As LC_25_	Pb LC_50_ + As LC_50_	Pb LC_50_ + As LC_75_
**Experiment 3**	Pb LC_75_ + As LC_25_	Pb LC_75_ + As LC_50_	Pb LC_75_ + As LC_75_

**Table 3:** Chi-square contrast of regression models for observed mixture exposure and concentration addition (CA) and independent action (IA) models.

Mixture Treatments	Observed vs. CA		Observed vs. IA	
	*x^2^*	*p*	*x^2^*	*p*
Pb LC_25_ + As LC_25_	22.427	0.0001	20.74	0.0001
Pb LC_25_ + As LC_50_	3.209	0.0733	-	-
Pb LC_25_ + As LC_75_	0.328	0.5666	-	-
Pb LC_50_ + As LC_25_	48	<0.0001	-	-
Pb LC_50_ + As LC_50_	50	<0.0001	50	0.0001
Pb LC_50_ + As LC_75_	33.333	<0.0001	-	-
Pb LC_75_ + As LC_25_	50	<0.0001	-	-
Pb LC_75_+ As LC_50_	25	<0.0001	-	-
Pb LC_75_ + As LC_75_	15.97	<0.0001	15.789	0.0001

## Data Availability

Data is available upon request.
